# Gold-Sensitized Silicon/ZnO Core/Shell Nanowire Array for Solar Water Splitting

**DOI:** 10.3389/fchem.2019.00206

**Published:** 2019-04-03

**Authors:** Fu-Qiang Zhang, Ya Hu, Rui-Nan Sun, Haoxin Fu, Kui-Qing Peng

**Affiliations:** Department of Physics and Beijing Key Laboratory of Energy Conversion and Storage Materials, Beijing Normal University, Beijing, China

**Keywords:** silicon nanowire, ZnO, core/shell heterostructure, solar water splitting, photosensitization

## Abstract

Solar water splitting represents one of the most promising strategies in the quest for clean and renewable energy. However, low conversion efficiency, use of sacrificial agents, and external bias for current water splitting system limit its practical application. Here, a gold-sensitized Si/ZnOcore/shell nanowire photoelectrochemical (PEC) cell is reported for efficient solar water oxidation. We demonstrated gold-sensitized n-Si/n-ZnO nanowire arrays exhibited higher energy conversion efficiency than gold-sensitized p-Si/n-ZnO nanowire arrays due to the favorable energy-band alignment characteristics. Without any assistance from an external electrical source and sacrificial reagents, gold-sensitized n-Si/n-ZnO core/shell nanowire array photoanode achieved unbiased water splitting under simulated solar light illumination. This method opens a promising venue to cost-efficient production of solar fuels.

## Introduction

Solar water splitting has a long history and continued to stimulate great interest in scientist around the world due to its promising application in storing the energy of the sun in the form of chemical fuels (Fujishima and Honda, 1972; Bard and Fox, 1995; Chen et al., 2010). As compared to water reduction reaction for hydrogen generation, water oxidation involves complex proton-coupled electron transfer process and the generation of oxygen is even more difficult. In the past decades, metal oxide semiconductors with wide band-gap have been widely explored as oxygen evolving photoanode due to their excellent stability in oxidizing environments. However, these wide band-gap semiconductors only absorb a small portion of sunlight, resulting in the poor water splitting efficiency. To achieve high efficient water splitting, sunlight has to be efficiently absorbed and utilized. Although significant efforts have been devoted to questing for cost-effective water splitting photocatalysts in the past decades (Wagner and Somorjai, 1980; Khaselev and Turner, 1998; Grätzel, 2001; Zou et al., 2001; Maeda et al., 2006; Grimes et al., 2008; Kanan and Nocera, 2008; Hwang et al., 2009; Kudo and Miseki, 2009; Lisorti et al., 2009; Yang et al., 2009; Sun et al., 2010; Walter et al., 2010; Ingram and Linic, 2011; Linic et al., 2011; Paracchino et al., 2011; Reece et al., 2011; Brillet et al., 2012; Warren and Thimsen, 2012; Qi et al., 2013; Wang et al., 2014; Liu et al., 2015; Yu et al., 2015), but the solar energy conversion efficiency reported is relatively low.

In this work, we demonstrate that this challenge may be addressed by devising an n-Si/n-ZnO core/shell nanowire heterojunction photoanode sensitized with gold nanoparticles (AuNPs). Without any assistance from an external electrical source and sacrificial reagents, such gold-sensitized n-Si/n-ZnO core/shell nanowires array photoanode shows efficient sunlight-driven water splitting ability. Moreover, such 3D dual-absorber water oxidation devices consist of earth-abundant materials can be prepared on an industrial scale with ease. The results also demonstrated that gold-sensitized n-Si/n-ZnO core/shell nanowiresexhibited higher energy conversion efficiency than gold-sensitized p-Si/n-ZnO core/shell nanowiresdue to reduced recombination of photo-generated charge carriers.

## Materials and Methods

**Figure 1:**
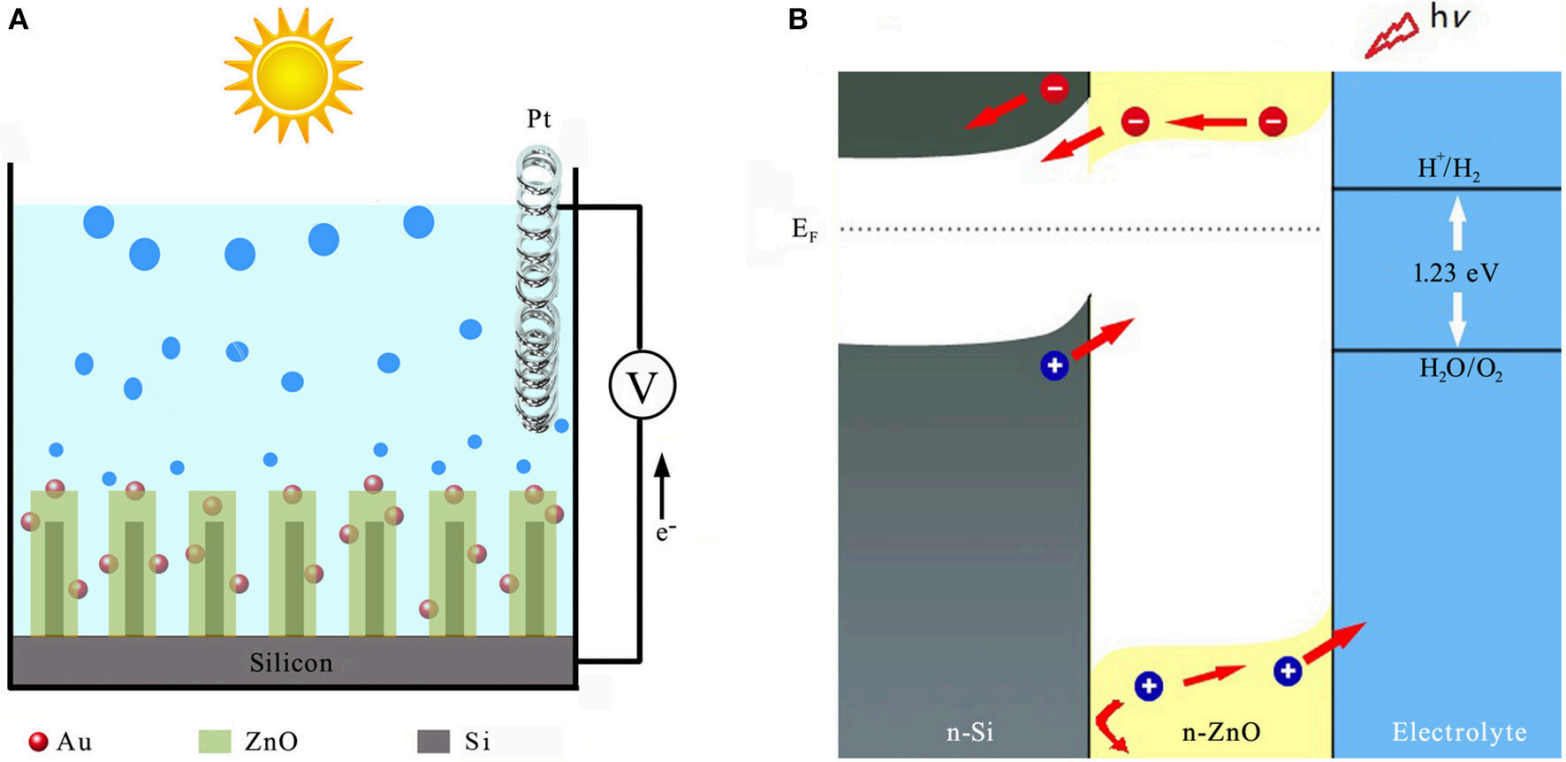
Structure and energy diagram of 3D n-SiNW/n-ZnO@AuNPs nanocomposite photoanode. **(A)** A schematic illustration of the nanostructured photoanode architecture. **(B)** Energy diagram for n-Si/n-ZnO photoanode in aqueous electrolyte under illumination.

Despite its relative instability, however, ZnO is adopted here due to its excellent electrical conductivity for efficient charge transport when compared to TiO_2_, WO_3_, and α-Fe_2_O_3_. Schematic illustration of the solar water splitting with n-Si/n-ZnO core/shell nanowire array sensitized with gold nanoparticles (AuNPs) is shown in Figure 1A. The n-Si/n-ZnO core/shell nanowire array significantly enhances light absorption over a wide solar spectral range, while reduces the photo-generated carrier loss due to the favorable energy-band alignment characteristics as illustrated in Figure 1B.

**Figure 2:**
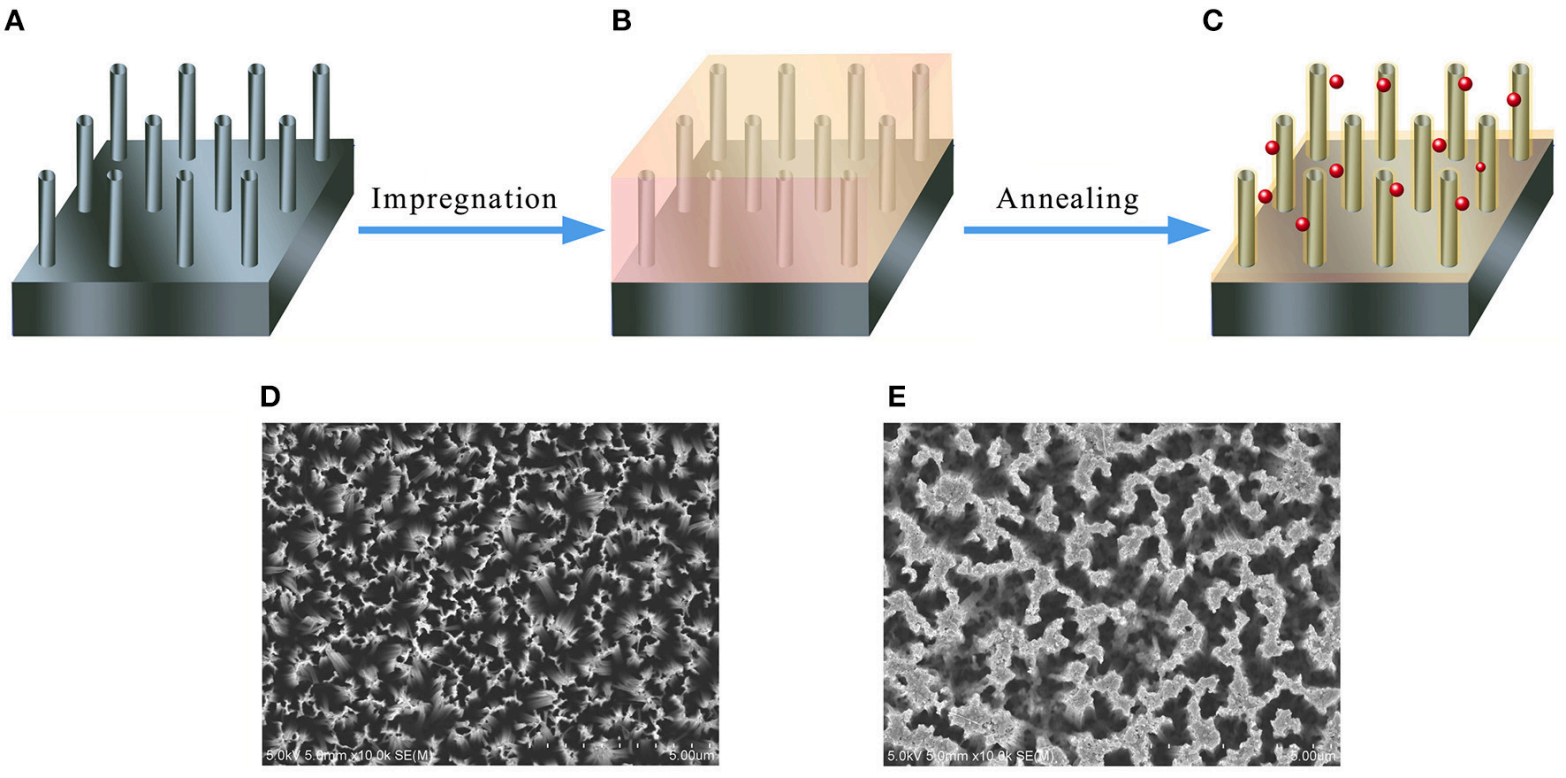
Schematic illustration of the fabrication process for 3D n-SiNW/n-ZnO@AuNPs nanocomposite photoanode and corresponding SEM images. **(A)** SiNW array prepared by metal-catalyzed etching of silicon. **(B)** SiNW array is impregnated with aqueous solutions containing Zn(NO_3_)_2_ and HAuCl_4_precursors. **(C)** n-Si/n-ZnO@AuNPs nanowires photoanode by annealing the impregnated SiNW array at 500°C in a vacuum tube furnace. **(D)** Top-view SEM image of n-SiNW array. **(E)** Top-view SEM image of n-Si/n-ZnO@AuNPs nanowires photoanode.

Figures 2A–C illustrate the fabrication process of the Si/n-ZnO core/shell nanowire heterojunction samples (for more details see the experimental section). Generally speaking, the silicon nanowire (SiNW) arrays are prepared by silver-catalyzed electroless etching of silicon wafer (Peng et al., 2002, 2003, 2006, 2008), then the SiNW arrays are impregnated with aqueous solutions containing mixed Zn(NO_3_)_2_ and HAuCl_4_ precursors. In the last, the Zn(NO_3_)_2_ and HAuCl_4_ wetted SiNW arrays are annealed in a vacuum tube furnace. The final n-SiNW/n-ZnO@AuNPs samples are yellowish black in color. The top-view scanning electron microscope (SEM) images of as-prepared SiNW array and n-SiNW/n-ZnO@AuNPs samples are shown in Figures 2D,E, respectively, showing arrays of dense nanowires vertically aligned on the silicon surfaces.

**Figure 3:**
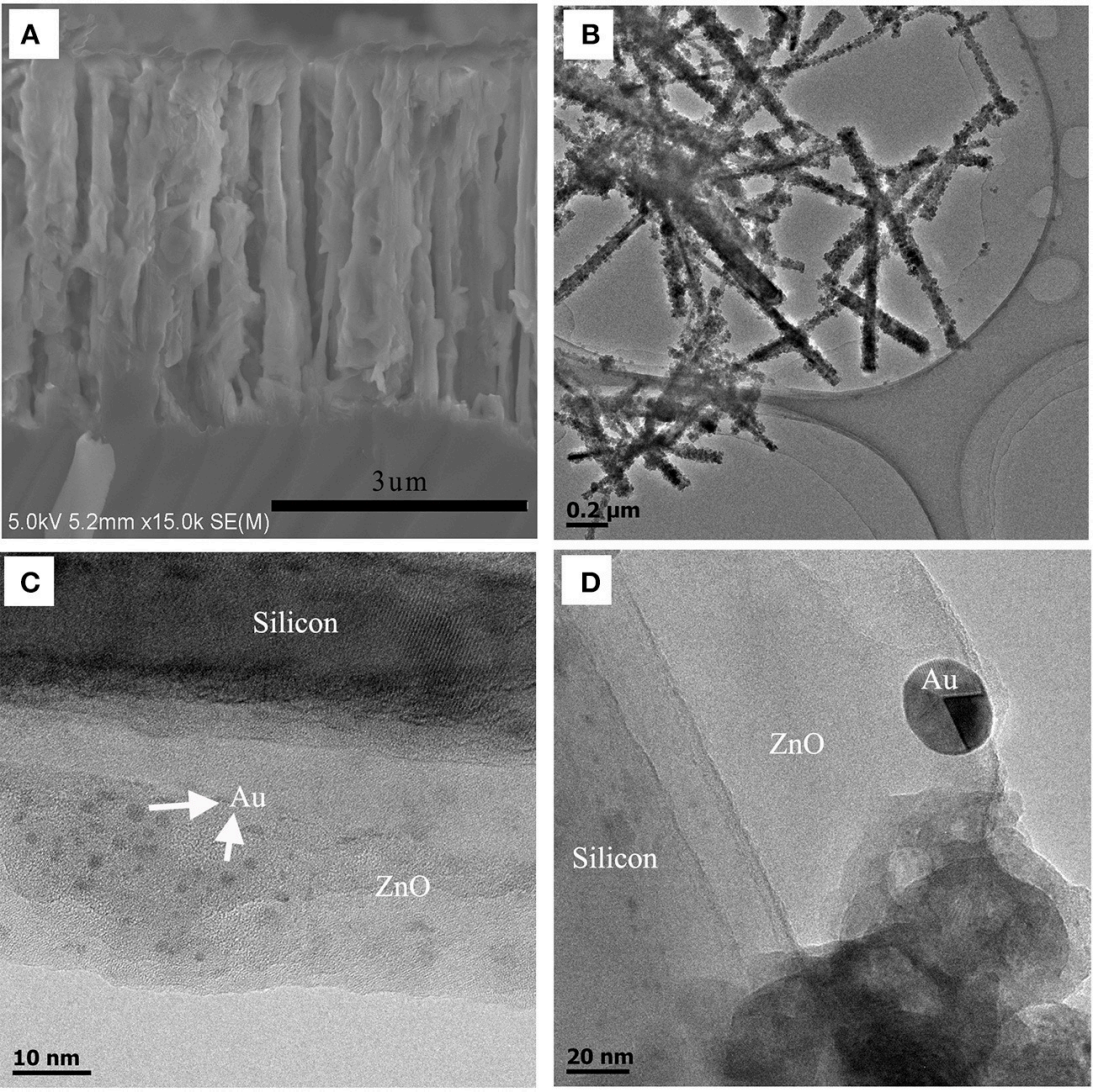
Structural characterization of Si/ZnO@AuNPs core/shell nanocomposite photoanode. **(A)** Side-view SEM image of Si/n-ZnO@AuNPs core/shell nanowire array. **(B)** Low-magnification TEM image of Si/n-ZnO@AuNPs core/shell nanowires. **(C,D)** High-magnification TEM images of Si/n-ZnO@AuNPs core/shell nanowire.

The side-view SEM image of the Si/n-ZnO@AuNPs core/shell nanowire arrays (Figure 3A) were characterized by transmission electron microscopy (TEM). Figures 3B–D shows the low-magnification and high-magnification TEM images of Si/n-ZnO@AuNPs core/shell nanowires. It can be clearly seen that the SiNWs are uniformly coated with crystalline ZnO particle layer. The thickness of the crystalline ZnO layer coated on the SiNW depends on the density of SiNWs and varied in the range from 20 to 60 nm. The AuNPs are between 2 and 20 nanometers in size, most of which are embedded within the ZnO layer while some of which are exposed to the surface of the ZnO layer. High-resolution TEM image of the edge of a single nanowire shown in Figure S1 clearly reveals the crystalline faceting at the Si/ZnO core/shell interface and the AuNPs loaded in the ZnO shell layer.

**Figure 4:**
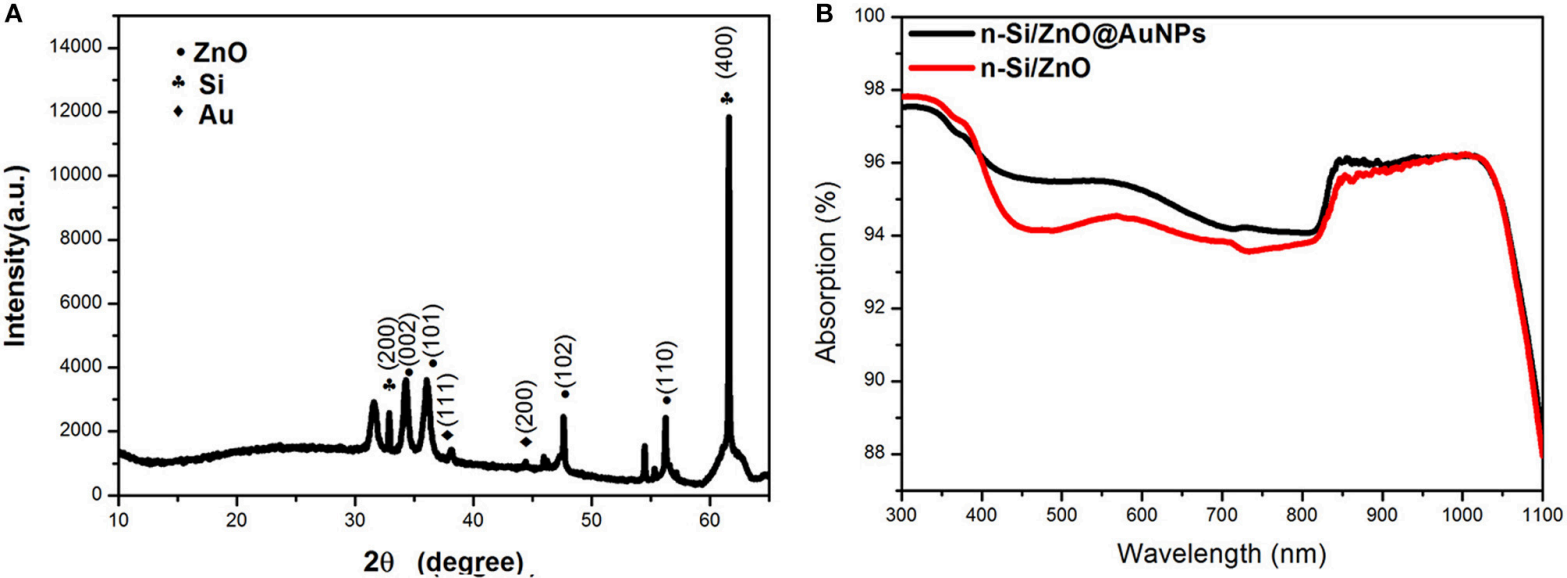
**(A)** XRD pattern of Si/n-ZnO@AuNPs core/shell nanowires photoanode. **(B)** Absorption spectra of Si/n-ZnO core/shell nanowire photoanodes with and without AuNPs.

Figure 4A shows the X-ray diffraction (XRD) pattern of the as-prepared Si/ZnO@AuNPs photoande. The diffraction peaks are well-matched with a mixture of the crystallographic structures of wurtzite zinc oxide, silicon, and gold. These results confirm that the solution immersion and annealing process is a facile route for large-scale fabrication of 3D composite photoelectrodes. The UV-visible absorption spectrum of as-prepared Si/n-ZnO@AuNPs core/shell nanowires photoanode is shown in Figure 4B, as a reference, the UV-visible absorption spectrum of Si/n-ZnO nanowires photoanode was included. It can be clearly seen that the Si/ZnO@AuNPs core/shell nanowires photoanode exhibits enhanced optical absorption in the wavelength range from 400 to 970 nm due to surface plasmon resonance (SPR) of AuNPs (Cushing et al., 2012; Li et al., 2013a,b). The enhanced light absorption due to SPR and photonic enhancement (Tian and Tatsuma, 2005; Li et al., 2013a) of AuNPs was further confirmed by electromagnetic simulation using finite-difference-time-domain (FDTD) method, as shown in Figure S2. The simulation geometry of the Si/ZnO@AuNPs core/shell nanowires photoanode is consistent with the experiment result, the diameter of AuNPs is 5 nm and the thickness of ZnO layer is 20 nm. Under the incident wavelengths of 496–636 nm of the light, the FDTD results show that the local electric fields near the AuNPs/n-ZnO interface are increased by about eight and 4 times, respectively. Thus, the generation rate of electron-hole pairs is expected to be greatly enhanced near the AuNPs/n-ZnO interface area due to the enhanced electric field intensity. In addition, the hot electrons due to the decay of plasmon transfer to the conduction band of ZnO result in photocurrent enhancement, as shown in Figure S3.

**Figure 5:**
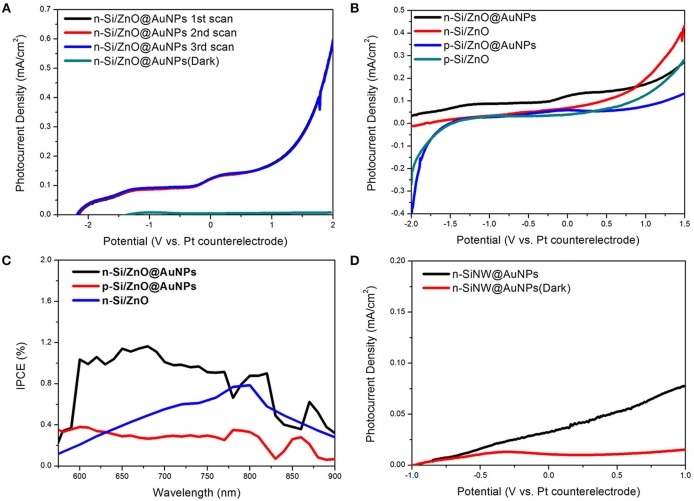
Photoelectrochemical properties of as-prepared nanocomposite photoanodes in 0.5 M Na_2_SO_4_ mild aqueous electrolyte. **(A)** The consecutive multiple linear scan curve of the composite n-Si/n-ZnO@AuNPs core/shell nanowires photoanode under illumination and dark. **(B)** Photocurrent density vs. potential characteristics of n-Si/n-ZnO@AuNPs, n-Si/n-ZnO, p-Si/n-ZnO@AuNPs, and p-Si/n-ZnO core/shell nanowire photoanodes under illumination, respectively. **(C)** IPCE spectra of n-Si/n-ZnO@AuNPs, n-Si/n-ZnO, and p-Si/n-ZnO@AuNPs core/shell nanowire photoanodes. The IPCE spectra are recorded at zero applied bias. **(D)** Photocurrent density vs. potential characteristics of n-SiNW@AuNPs photoanode under illumination and in dark.

The photoelectrochemical (PEC) properties of the sample were characterized in a two-electrode configuration with platinum wire coil as the counter electrode. A 0.5 M Na_2_SO_4_ mild aqueous solution (PH~7) served as the electrolyte and no sacrificial reagents were used. All the tests were conducted under a.m. 1.5 G illumination with an intensity of 100 mW/cm^2^. Figure 5A shows the consecutive multiple linear scan curve of the composite n-Si/n-ZnO@AuNPs core/shell nanowires photoanode under illumination and dark. The little difference of the curves in the linear scan indicate the repeatability and stability of the photoanode in solution. Moreover, the n-Si/n-ZnO@AuNPs core/shell nanowires photoanode shows 0.115 mA cm^−2^ photocurrent density at 0 V, implying water oxidation activity without any assistance from an external electrical source. Moreover, the photocurrent density of the photoanode shows progress compared with previous results under zero applied bias (Lin et al., 2012; Guo et al., 2013), as shown in Table S1. We also use a three-electrode system to investigate the onset potential of the n-Si/ZnO@AuNPs photoanode for water oxidation as shown in Figure S4. It can be seen the onset potential in three-electrode system has about 1.1 V potential shift compare with two-electrode system, which means 1.71 V shift vs. RHE. The current-potential characteristics of the n-Si/n-ZnO, p-Si/n-ZnO@AuNPs and p-Si/n-ZnO core/shell nanowire photoanodes were also measured for comparison as shown in Figure 5B. The dark current density of these photoanode is shown in Figure S5. The dark current densities are negligible as compared to the photocurrent densities under illumination, revealing few chemical reactions occurred in dark. Under simulated solar illumination, the photocurrent density of n-Si/n-ZnO core/shell nanowires photoanode at 0 V is 0.064 mA cm^−2^. The photocurrent density is about two times low and the open-circuit potential is higher as compared to the n-Si/ZnO@AuNPs core/shell nanowires photoanode. We suggest the AuNPs trigger the unbiased solar water splitting through surface plasmon resonance (SPR), plasmons decay induced hot electrons transfer and the plasmon resonance energy transfer (PRET). The PRET decreases the distance of the holes travel to the electrolyte and therefore improve the photocurrent density. We note that the photocurrent density at 0 V of p-Si/n-ZnO@AuNPs and p-Si/n-ZnO core/shell nanowire photoanodes is 0.042 mA cm^−2^ and 0.014 mA cm^−2^, respectively. More interestingly, the PEC water oxidation performance of n-Si/n-ZnO@AuNPs core/shell nanowires photoanode is higher than that of the p-Si/n-ZnO@AuNPs core/shell nanowires photoanode. This could be understood by the band bending characteristics of n-Si/n-ZnO (Figure S6a) and p-Si/n-ZnO junctions (Figure S6b). In p-Si/n-ZnO junctions, the photo generated holes in ZnO layer can move either to the p-Si or to the electrolyte for water oxidation. In contrast, the holes in the ZnO layer of n-Si/n-ZnO junctions can only move to the electrolyte and the electrons in the silicon move to the electrode for a circuit.

The sunlight-driven water splitting performance of as-prepared composite photoanodes was further evaluated by the incident photo-to-current efficiency (IPCE). Figure 5C shows the IPCE spectra as a function of wavelength recorded at zero bias for n-Si/n-ZnO@AuNPs, n-Si/n-ZnO and p-Si/n-ZnO@AuNPs core/shell nanowire photoanodes. It is clearly observed that the n-Si/n-ZnO@AuNPs core/shell nanowire photoanode shows enhanced photoresponse in a wide wavelength range. In contrast, the photoresponse of p-Si/n-ZnO@AuNPs core/shell nanowires photoanode is relatively low in the same wavelength range due to the unfavorable energy-band alignment characteristics. The IPCE difference between n-Si/n-ZnO@AuNPs and n-Si/n-ZnO photoanode further confirms the effect of AuNPs. In order to certify the role of ZnO layer in improving the performance of n-Si/n-ZnO@AuNPs core/shell nanowires photoanode for water oxidation, we conducted the current-potential measurement of n-SiNW@AuNPs photoanode in 0.5 M Na_2_SO_4_ solution. Figure 5D shows the current-potential characteristics of n-SiNW@AuNPs photoanode under illumination and dark. The photocurrent density at 0 V is 0.02 mA cm^−2^, which quantitatively shows the role of ZnO layer in promoting the performance of n-Si/n-ZnO@AuNPscore/shell nanowire photoanode.

The influence of the position of AuNPs in the ZnO layer upon the water splitting performance is evaluated. Figure S7 illustrates the n-Si/ZnO core/shell nanowires photoanodes decorated with AuNPs in “mixed,” “outer,” and “inner” configurations. Figure S8 shows the J-E curves of the n-Si/ZnO core/shell nanowires decorated with AuNPs in different position. All the tests are recorded in 0.5 M Na_2_SO_4_ solution. The photocurrent density at 0 V for the photoanodes decorated with AuNPs in outer and inner configurations are 0.073 and 0.080 mA/cm^2^, respectively. The results clearly show that the nanowires photoanode decorated with AuNPs in mixed configuration exhibited better water splitting performance. The suppressed plasmonic photosensitization for AuNPs in inner configuration limits the catalytic effect. And the lower electrical conductivity in the surface configuration limits the separation of electron-hole pairs. Such distinct performance implies that AuNPs play the roles of plasmonic photosensitization, co-catalyst for water splitting reaction, and electrical conductivity enhancement.

**Figure 6:**
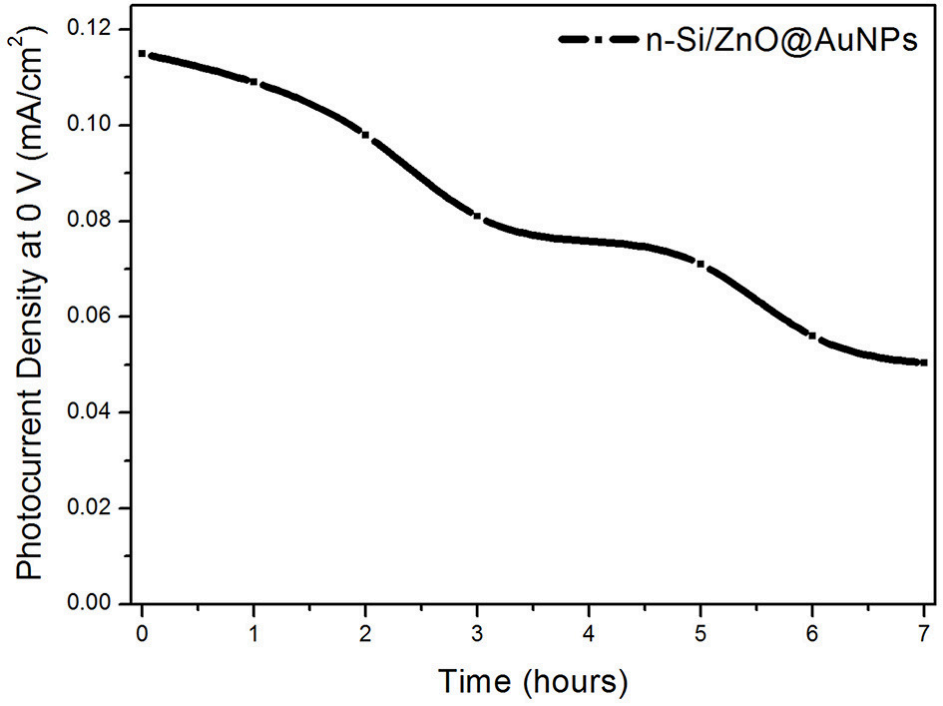
Photocurrent evolution vs. illumination time of the n-Si/n-ZnO@AuNPs core/shell nanowires photoanode in 0.5 M aqueous Na_2_SO_4_ solution.

The stability of the n-Si/n-ZnO@AuNPs core/shell nanowires photoanode was assessed by measuring the photocurrent density at 0 V in 0.5 M Na_2_SO_4_ solution under a.m. 1.5 G illumination of 100 mW cm^−2^. Figure 6 shows the evolution of photocurrent density of n-Si/n-ZnO@AuNPs core/shell nanowires photoanode at 0 V with the illumination time. The photocurrent density gradually declined to ~70% of its initial value over 3 h. This is a good stability performance in comparison with previous reports (Qiu et al., 2012).

## Conclusions

In summary, we have demonstrated an efficient solar water splitting system based on n-Si/n-ZnO core/shell nanowire array photosensitized with AuNPs. The n-Si/n-ZnO@AuNPs core/shell nanowires photoanode demonstrate much higher efficiency than p-Si/n-ZnO@AuNPs core/shell nanowires photoanode. We suggest that the ZnO shell and incorporated AuNPs play crucial catalytic and plasmonic photosensitization roles, while silicon core absorbs light and generates photocarriers. AuNPs also may function as efficient co-catalyst for water splitting reaction. We believe such solar water splitting system represents a step toward the goal of cost-effective large-scale production of solar fuels.

## Author Contributions

All authors listed have made substantial, direct and intellectual contributions to the work: F-QZ performed all experiments and wrote the manuscript. YH, R-NS, and HF prepared the samples and analyzed the data. K-QP analyzed the data and wrote the manuscript. All authors discussed, reviewed and approved the manuscript.

### Conflict of Interest Statement

The authors declare that the research was conducted in the absence of any commercial or financial relationships that could be construed as a potential conflict of interest.
