# Supplementary material for: Gold-Sensitized Silicon/ZnO Core/Shell Nanowire Array for Solar Water Splitting
Source: Front Chem. 2019 Apr 3;7:206. doi: 10.3389/fchem.2019.00206 (PMC6456691; doi:10.3389/fchem.2019.00206)
Supplement: Supplementary file 1 [file Data_Sheet_1.pdf]

Supplementary Material

Gold-sensitized Silicon/ZnO Core/shell Nanowire Array for Solar Water splitting

Fu-Qiang Zhang, Ya Hu, Rui-Nan Sun, Haoxin Fu, Kui-Qing Peng*

*** Correspondence:** Kui-Qing Peng: kq_peng@bnu.edu.cn

Fabrication of Silicon Nanowires Arrays. Large-scale aligned single-crystalline silicon nanowire (SiNW) arrays are prepared on Si wafers by means of metal-catalyzed electroless etching (MCEE) method in HF-$AgNO_3$ solution. 2×2 cm^2 n-type Si (100) (P doped, 2.0-2.7 Ω cm) and p-type Si (100) (B doped, 1-10 Ω cm) pieces are firstly ultrasonically degreased in acetone and ethanol for 10 minutes, respectively, and then immersed in a boiling solution of H_2SO_4/H_2O_2 for 30 minutes. After thoroughly rinsed with DI water, the Si pieces are immersed in mixed aqueous HF/$AgNO_3$ solution (0.25M $AgNO_3$, 40% HF solution) for etching. The produced SiNWs samples are immersed in concentrated nitric acid (65%) for 30 min to remove residual Ag. After removing the oxide layer with diluted HF solution (1.0%), the samples were dried at room temperature.

Composite Photoanode Fabrication. For the Si/ZnO core/shell nanowires photoanode decorated with AuNPs, the cleaned SiNW arrays are firstly fully wetted in aqueous solution containing $Zn(NO_3)_2$ (4.34mol/L) and $HAuCl_4$ precursors (0.048 mol/L) for 50 minutes. Then the wetted SiNW arrays are dried in vacuum at 70°C for 1 hour and subsequently annealed in a vacuum tube furnace at 500°C for 5 hours. For the Si/ZnO core/shell nanowires photoanode, the cleaned SiNW arrays are fully wetted in aqueous solution containing $Zn(NO_3)_2$ (4.34 mol/L) for 50 minutes. Then the wetted SiNW arrays are dried in vacuum at 70°C for 1 hour and subsequently annealed in a vacuum tube furnace at 500°C for 5 hours. For the SiNW photoanode only decorated with AuNPs, the surface of SiNWs are coated with AuNPs by annealing $HAuCl_4$ (0.048 mol/L) wetted SiNWs at 500°C for 2hours. For the photoanode decorated with AuNPs in outer configuration, ZnO shell layer was firstly prepared by thermal annealing $Zn(NO_3)_2$ (4.34 mol/L) wetted SiNWs at 500°C for 5 hours and then coated AuNPs by thermal annealing $HAuCl_4$ (0.048 mol/L) wetted Si/ZnO core/shell nanowires at 500°C for 2h. For the photoanode decorated with AuNPs in inner configuration, the surfaces of SiNWs are firstly coated with AuNPs by annealing $HAuCl_4$ (0.048 mol/L) wetted SiNWs at 500°C

for 2h and then coated with ZnO shell layer by thermal annealing $Zn(NO_3)_2$ (4.34 mol/L) wetted SiNWs at 500°C for 5 hours.

Structural and Photocatalytic Activity Test. The structures of as-prepared 3D nanostructure photoanodes were investigated using a field emission scanning electron microscopy (SEM, HITACHI S-4800), a transmission electron microscopy (TEM, JEOL JEM-2100), a UV-Vis-NIR spectrophotometer (Cary-5000, Agilent, US) and X-ray diffraction using Cu Kα radiation(Rigiku, Japan). Under the AM 1.5G illumination with an intensity of 100 mW/cm^2, the photoelectrochemical measurements were performed in an electrochemical cell with a 1.0 cm^2 quartz window. The electrolyte is 0.5 M Na_2SO_4 aqueous solution (PH~7) without introducing any sacrificial reagents. Ohmic contact is made by scratching high-purity In-Ga alloy on the rear side of Si substrate. The light source was a 500W Xe lamp. In the three-electrode system test, the sample served as the working electrode, Pt wire served as the counter electrode and the Ag/AgCl with 3 M KCl solution as the reference electrode. The incident photo-to-current efficiency (IPCE) was measured using QEX 10 measurement system (PV Measurements).

Supplementary Figures

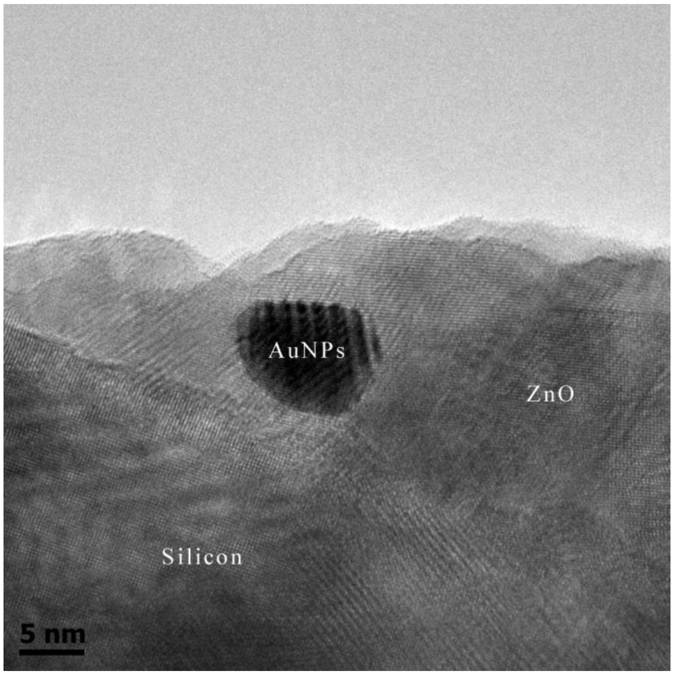

Supplementary Figure 1 High-resolution TEMimageof the edge of an n-Si/ZnO core/shell nanowire loaded with a gold nanoparticle.

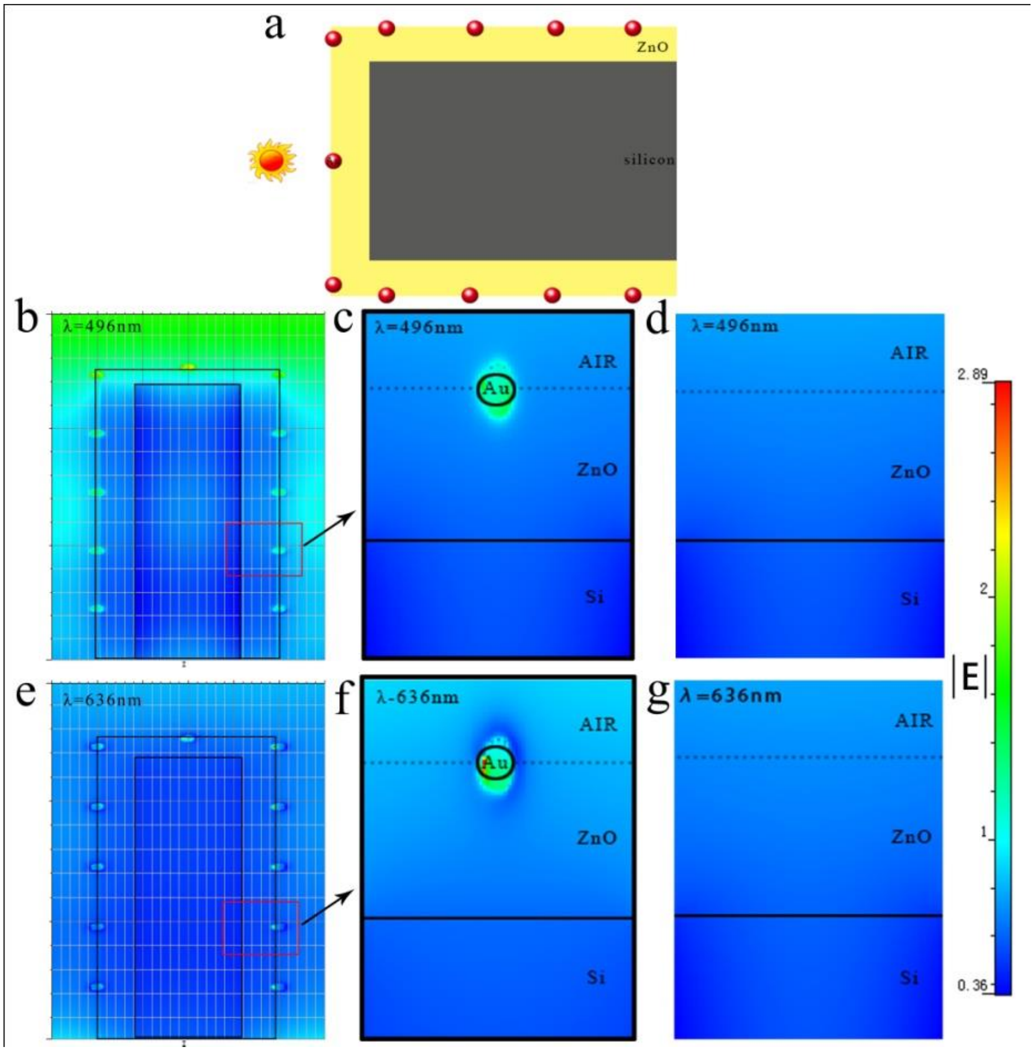

Supplementary Figure 2 Electromagneticsimulation of Si/ZnO@AuNPs and Si/ZnOcore/shell nanocomposite photoanode. **(a)** Simulation geometry of Si/ZnO@AuNPscore/shell photoanode.**(b, e)**Local electric field enhancement in ZnOlayerdue to surface plasmonresonanceof AuNPs under the incident wavelengths of 496 and 636 nm, respectively. (**c, f**) Magnified electric field distribution in the red box region under the incident wavelengths of 496 and 636 nm, respectively. **(d, g)** Magnified electric field distribution in the red box region without AuNPsunder the incident wavelengths of 496 and 636 nm, respectively.

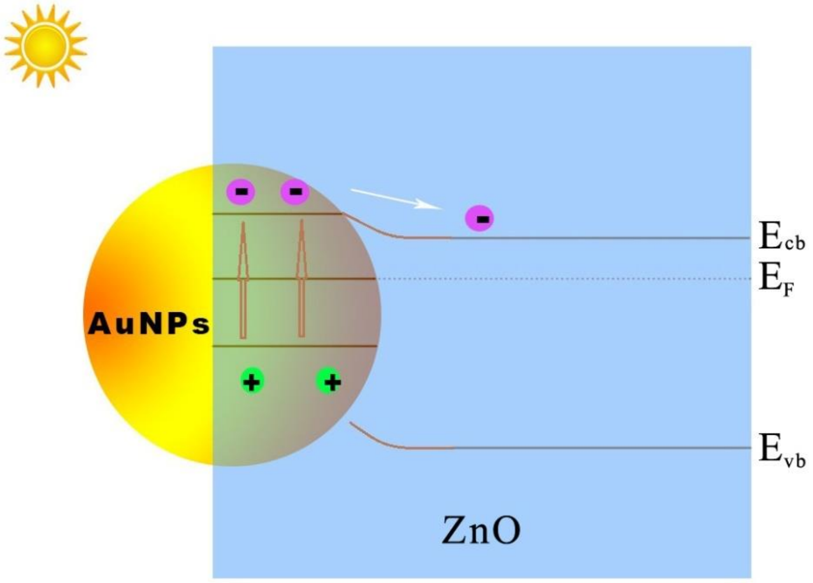

Supplementary Figure 3 Schematicenergy band diagram illustrating the transport of hot electrons produced by plasmon decay into conduction band of ZnOover theAu/ZnO contact barrier.

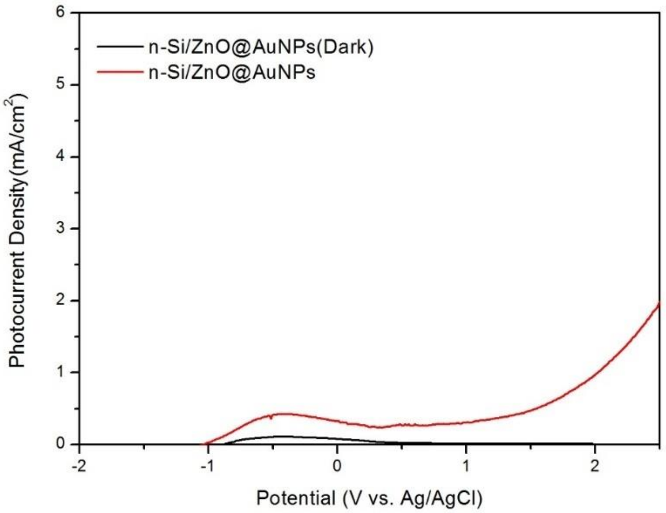

Supplementary Figure4 J-E curves of n-Si/ZnO@AuNPsphotoanode in three-electrode electrochemical system with Ag/AgCl as reference electrode.

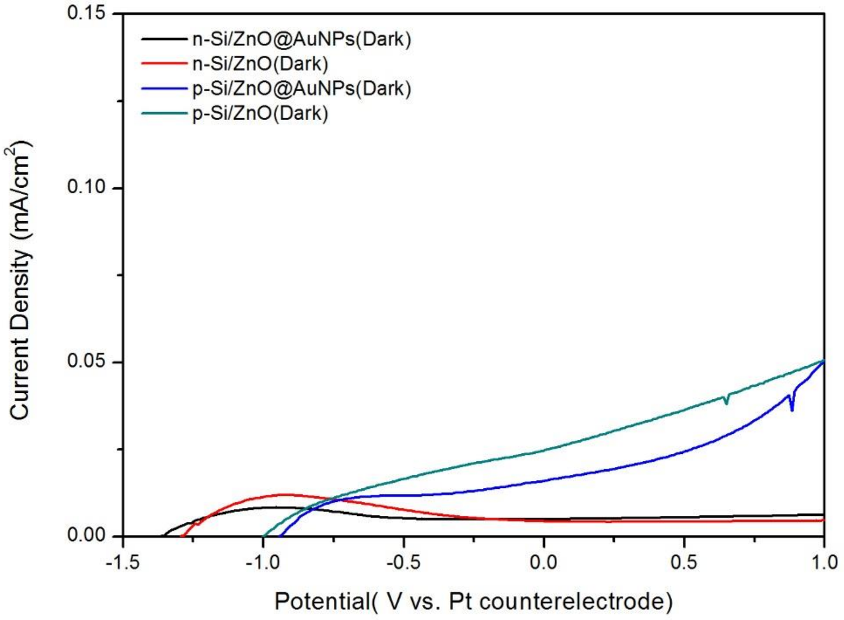

Supplementary Figure5 Dark current density of the n-Si/n-ZnO@AuNPs, n-Si/n-ZnO, p-Si/n-ZnO@AuNPs and p-Si/n-ZnO core/shell nanowire photoanodes, respectively.

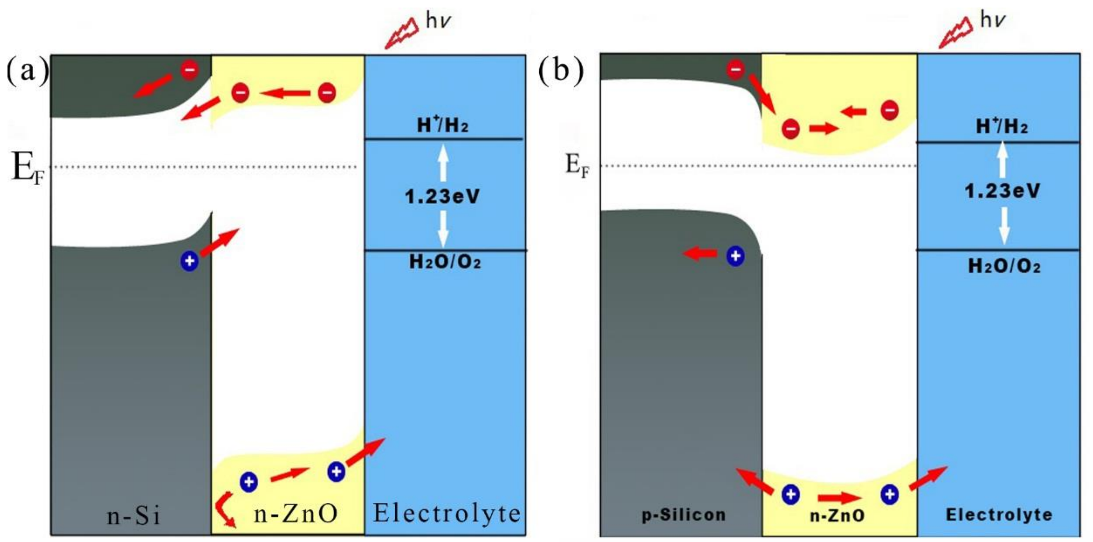

Supplementary Figure6 Energy band diagram scheme of the (a) n-Si/n-ZnO and (b) p-Si/n-ZnO core/shell nanowire photoanode in electrolyte under illumination.

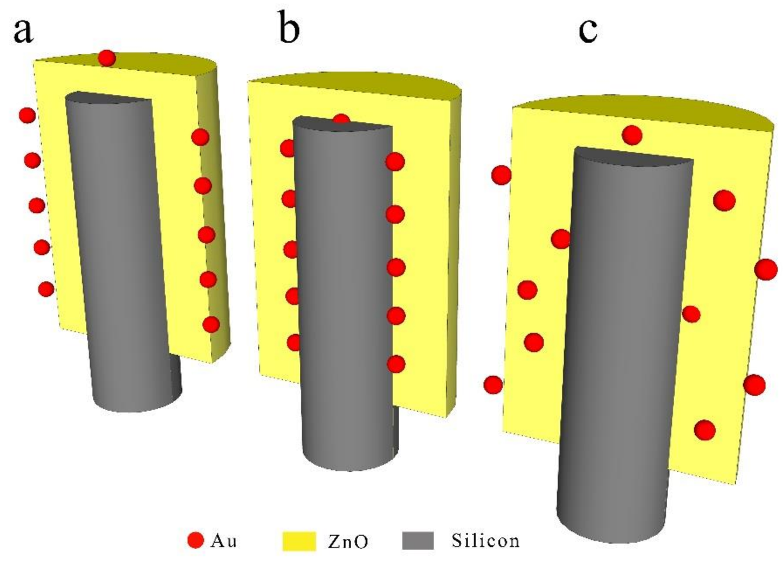

Supplementary Figure7 Schematic illustration of the Si/ZnO core/shell nanowire photoanodes decorated with AuNPs in “outer”, “inner” and “mixed” configurations.

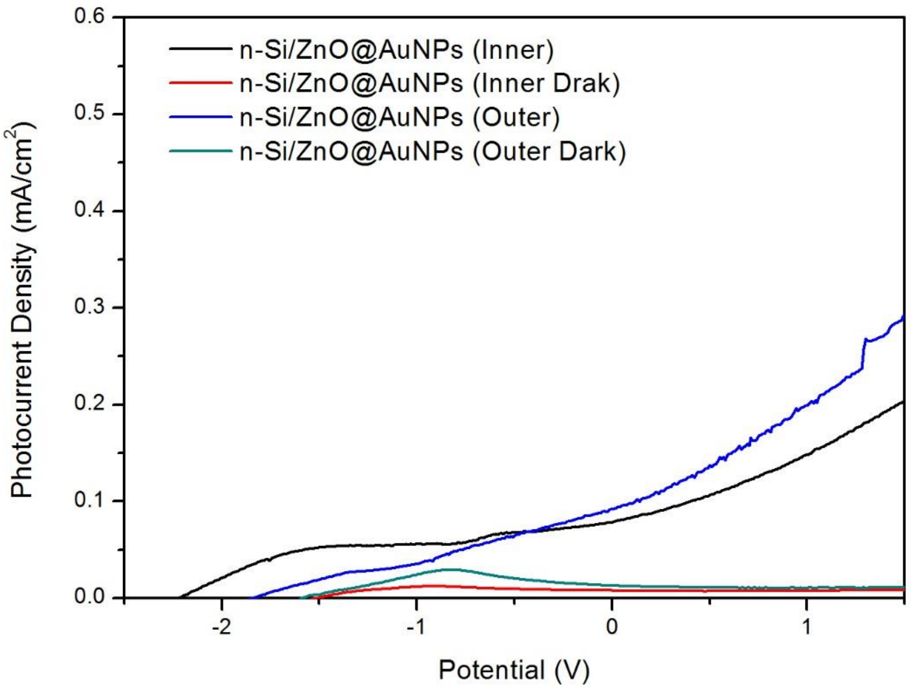

Supplementary Figure8 J-E curves of the n-Si/ZnO core/shell nanowires decorated with AuNPs in “inner” and “outer” configurations.

Structure	Electrolyte	Electrode	Applied Potential (V)	Photocurrent Density (mA/cm^2)
n-SiNW/ZnO@AuNPs	0.5M Na_2SO_4	Pt Counter Electrode	0	0.115
$Ag@Ag_3(PO_4)_{1-x}$ photosensitized ZnO nanorod	0.5M Na_2SO_4	Pt Counter Electrode	0	~0.1
Graphene Quantum Dots Sensitized ZnO Nanowire	0.5M Na_2SO_4	Pt Counter Electrode	0	~0.06

Supplementary Table 1. The photocurrent density of n-SiNW/ZnO@AuNPs photoanode compared with previous result under zero applied bias.
